# Effect of Bone Marrow-Derived Mesenchymal Stem Cells on Endotoxin-Induced Oxidation of Plasma Cysteine and Glutathione in Mice

**DOI:** 10.4061/2010/868076

**Published:** 2010-03-08

**Authors:** Smita S. Iyer, Edilson Torres-Gonzalez, David C. Neujahr, Mike Kwon, Kenneth L. Brigham, Dean P. Jones, Ana L. Mora, Mauricio Rojas

**Affiliations:** ^1^Nutrition and Health Sciences Program, Emory University, Atlanta, GA 30322, USA; ^2^Center for Translational Research in the Lung, Emory University, Atlanta, GA 30322, USA; ^3^Clinical Biomarkers Laboratory, Emory University, Atlanta, GA 30322, USA; ^4^Division of Pulmonary, Allergy, and Critical Care Medicine, Emory University, Atlanta, GA 30322, USA; ^5^McKelvey Center for Lung Transplantation and Pulmonary Vascular Diseases, Emory University, Atlanta, GA 30322, USA

## Abstract

Bone marrow-derived mesenchymal stem cells (BMDMSC) are emerging as a therapeutic modality in various inflammatory disease states, including acute lung injury (ALI). A hallmark of inflammation, and a consistent observation in patients with ALI, is a perturbation in the systemic redox environment. However, little is known about the effects of BMDMSC on the systemic redox status. The objective of the present study was to determine whether exogenously infused BMDMSC protect against endotoxin-induced oxidation of plasma cysteine (Cys) and glutathione (GSH) redox states. To determine the effect on the redox state if BMDMSC, mice received endotoxin intraperitoneally (1 mg/kg), followed by intravenous infusion of either 5 × 10^5^ BMDMSC or an equal volume of saline solution. Control mice received intraperitoneal endotoxin followed by 5 × 10^5^ lung fibroblasts given intravenously. Cys, cystine (CySS), GSH, and glutathione disulfide (GSSG) concentrations were determined by HPLC. Results showed sequential preservation of plasma Cys and GSH levels in response to BMDMSC infusion. The data show that BMDMSC infusion leads to a more reducing Cys and GSH redox state. The findings are the first to demonstrate that BMDMSC have antioxidant effects in vivo, and add to our understanding of the systemic effects of BMDMSC in lung injury.

## 1. Introduction

The inflammatory response to pathogens, physical trauma, or toxic stimuli is critical in host defense, but excessive and unregulated inflammation can injure the lungs [1]. In patients with gram negative sepsis, a disregulated inflammatory response to bacterial endotoxin increases the risk for acute lung injury (ALI), which can lead to severe respiratory failure termed the acute respiratory distress syndrome (ARDS) [1]. ALI and ARDS are associated with significant morbidity and mortality [2]. Consequently, strategies to attenuate the inflammatory response in ALI and ARDS are of considerable interest.

An emerging therapeutic modality in various inflammatory diseases is the use of bone marrow-derived mesenchymal stem cells/mesenchymal stromal cells (BMDMSCs) [3]. BMDMSCs are multipotent cells that can be isolated from the bone marrow and expanded in culture relatively easily. Several studies, including our own, have shown that exogenously infused BMDMSCs protect against endotoxin-induced inflammation, and ALI in mice [4–6]. In these studies, the protective effects of BMDMSCs are mediated by a decrease in circulating proinflammatory cytokine levels, and appear to be independent of BMDMSCs engraftment into the lung. A hallmark of inflammation, and a consistent observation in patients with ALI, is a perturbation in the extracellular thiol/disulfide redox environment. However, little is known about the effects of BMDMSCs on the systemic thiol/disulfide redox environment. 

Cysteine (Cys) and its disulfide cystine (CySS), together with glutathione (GSH) and glutathione disulfide (GSSG), comprise the major extracellular thiol/disulfide redox control systems. Cys and GSH are important determinants of cytokine expression, and alteration in Cys and GSH metabolism is a central feature of inflammation [7]. Because the thiol/disulfide redox environment is intimately linked to inflammation and tissue injury, the present study was undertaken to examine the effects of exogenous BMDMSC infusion on plasma Cys and GSH levels. Accumulating evidence shows that the redox states of Cys and GSH are independently regulated [8]; therefore a secondary purpose was to determine whether the two redox couples respond differently to BMDMSC infusion. Mice received endotoxin intraperitoneally, followed by intravenous infusion of 5 × 10^5^, CD 45-immunodepleted BMDMSCs. Results showed sequential preservation of plasma Cys and GSH redox states in response to BMDMSC infusion. The data provide the novel observation that BMDMSC infusion modulates thiol/disulfide redox status in vivo.

## 2. Materials and Methods

### 2.1. Materials

Except as indicated, all chemicals were purchased from Sigma Chemical Corporation (Sigma, St. Louis, MO). Distilled, deionized water was used for analytical purposes. HPLC quality solvents were used for HPLC.

### 2.2. Experimental Animals

Experiments were conducted using 10–14-week-old, female C57BL/6J mice (Jackson Laboratories, Bar Harbor, ME). Mice were housed in cages and maintained on a 12-hour light-12-hour dark cycle at the Division of Animal Resources at Emory University. All animals were fed pelleted rodent food (Test Diet 5015, Lab Diet Inc., Richmond, IN) and had free access to water. All experiments were initiated during the light cycle. All animal protocols were reviewed and approved by the Institutional Animal Care and Use Committee. Because estrogens may exert anti-inflammatory effects, influence of estrous cycle cannot be entirely ruled out in the current experiments [9].

### 2.3. LPS Administration

Escherichia coli O55 : B5 LPS, dissolved in sterile PBS, was administered intraperitoneally at a dose of 1 mg LPS/kg body weight. Mice were subsequently anesthetized by isofluorane inhalation (Baxter Pharmaceuticals, Deerfield, IL), and given 5 × 10^5^ BMDMSC in 0.1 mL PBS, 5 × 10^5^ primary lung fibroblasts in 0.1 mL PBS, or 0.1 mL PBS. The retro-orbital vein was used for intravenous administration. Studies by group have shown that BMDMSCs at this dose exert anti-inflammatory effects in vivo [10]. Therefore, this dose was used to determine if BMDMSCs also exert protective effects on oxidative stress. Animals were sacrificed at 2, 6, and 24 hours postLPS administration. No mortality was associated with LPS administration.

### 2.4. Sample Collection and Analysis of Cys, CySS, GSH, and GSSG

Samples were collected using a method optimized to minimize errors due to collection and processing [11]. Mice were anesthetized by isofluorane inhalation and blood was collected by submandibular bleeding using a 4 mm mouse-bleeding lancet (Medipoint, Inc. Mineola, NY). To minimize artificial overestimation of GSH due to hemolysis, blood was collected into a heparin-coated eppendorf tube to inhibit coagulation. Samples that showed evidence of hemolysis were not included in the analysis. 

The collected blood was (0.18 mL) immediately transferred to an eppdendorf tube containing 0.02 mL of preservation solution. The preservation solution included heparin, serine-borate to inhibit degradation of GSH by *γ*-glutamyltranspeptidase, bathophenanthroline disulfonate to inhibit oxidation of GSH and Cys, and iodoacetic acid to alkylate GSH and Cys. To facilitate quantification of the thiols and disulfides, *γ*-glutamyl glutamate (*γ*-Glu-Glu) was used as an internal standard [12]. Samples were centrifuged at 16000 g for 60 seconds to remove precipitated protein, and 0.1 mL of the supernatant was immediately transferred to an equal volume of ice-cold 10% (w/v) perchloric acid. Samples were immediately stored at −80°C and were derivatized with dansyl chloride within 1 month. Stability tests have shown that nonderivatized samples are stable for at least 2 months at −80°C [11]. 

For HPLC analysis (Gilson Medical Electronics, Middleton, WI), derivatized samples were centrifuged, and 35 *μ*l of the aqueous layer was applied to the Supercosil LC-NH_2_ column (25 cm × 4.6 mm; Supelco, Bellefunk, PA). Derivatives were separated with a sodium acetate gradient in methanol/water and detected by fluorescence [11]. Concentrations of thiols and disulfides were determined by integration relative to the internal standard. Redox states (*E*_*h*_) of the GSH/GSSG, and Cys/CySS pools, given in millivolts (mV), were calculated from concentrations of GSH, GSSG and Cys, CySS in molar units with the following forms of the Nernst equation for pH 7.4 : GSH/GSSG, *E*_*h*_ = −264 + 30  log ([GSSG]/[GSH]^2^), Cys/CySS, *E*_*h*_ = −250 + 30log   ([CySS]/[Cys]^2^) [13].

### 2.5. Cell Culture

Cells were maintained in a humidified 5% CO_2_ incubator at 37°C. A frozen vial of murine bone marrow-derived mesenchymal stem cells, obtained from the Tulane Center for Gene Therapy, New Orleans, Louisiana, was thawed and expanded as previously described [14]. BMDMSCs were propagated in a 175 cm^2^ flask in Iscove's modified Dulbeccos's medium (IMDM) containing 9% fetal bovine serum (FBS, Atlanta, Biologicals, Norcross, GA), 9% horse serum, and 1% penicillin-streptomycin Cells were harvested at 70% confluency using 0.25% trypsin. Harvested cells were depleted of contaminating hematopoietic cells by magnetic immunodepletion with anti-CD45 conjugated to phycoerythrin (PE), followed by PE-conjugated magnetic beads (Miltenyi Biotec, Auburn, CA). After negative depletion, plastic-adherent cells were propagated for upto 10 passages. For in vivo infusion, cells were detached using 0.25% trypsin at 37°C for 5 minutes. Trypsin was neutralized by adding IMDM with serum. The cell suspension was centrifuged and resuspended in sterile PBS (without Ca and Mg). After two additional washes with PBS, cells were counted by trypan blue exclusion and were resuspended at 5 × 10^5^ cells per 0.1 mL of PBS. 

Primary fibroblasts were isolated from the lungs of 8–10-week-old wild type C57BL/6J mice, as previously described [15]. Cells were maintained in DMEM supplemented with 10% FBS and 1% penicillin-streptomycin (MediaTech, Manassas, VA) for up to 10 passages. For in vivo infusion, cells were harvested and processed as described above. Passage number of BMDMSC and primary fibroblasts was similar in all animals for all time points within each experimental replicate.

### 2.6. Cytokine Analysis

Levels of IL-1*β*, TNF-a, and IFN-y in plasma were detected by ELISA (R&D Systems, Minneapolis, MN).

### 2.7. Flow Cytometry

Flow cytometry for expression of a panel of surface markers was performed on a FACScan cytometer (Becton Dickinson) using standard techniques. BMDMSCs were harvested by trypsinization, washed in PBS, and stained with the following antibodies : Fluorescein isothiocyanate- (FITC-) anti-CD45, PE-anti-CD11b, and PerCP-Cy5.5-anti-Sca-1 (Pharmigen San Jose, CA). Acquired data were analyzed using FlowJo software (Tree Star, San Carlos, CA).

### 2.8. Differentiation Assays

The ability of BMDMSC to differentiate into multiple mesenchymal lineages was determined using a mesenchymal stem cell functional identification kit (R&D Systems Minneapolis, MN USA). Adipogenic differentiation was induced by seeding cells for 14 days in *α*-MEM medium with 10% FBS, 1% penicillin-streptomycin, and adipogenic supplement containing hydrocortisone, isobutylmethylxanthine, and indomethacin. Oil droplets in the cultures were identified by staining cells with Oil Red O. Osteogenic differentiation was induced by culturing cells for 21 days in *α*-MEM medium with 10% FBS, 1% penicillin-streptomycin, and osteogenic supplement containing dexamethasone, ascorbate-phosphate, and *β*-glycerolphosphate. The calcium containing precipitates were visualized after staining with 2% Alizarin red adjusted to a pH of 4.4 with ammonium hydroxide. Chondrogenic differentiation was induced by pelleting cells in a 6-well plate and culturing in D-MEM/F-12 with 1% penicillin-streptomycin, and chondrogenic supplement containing dexamethasone, ascorbate-phosphate, proline, pyruvate, and TGF-*β*3. Pelleted cells were cultured for 14 days and stained with Alcian blue to visualize acid mucopolysachharides.

### 2.9. Statistical Methods

Data are presented as means + SEM from 4–6 animals. Statistical analysis was done using Graph Pad Prism v 4.01. A one-tailed, unpaired *t*-test was used to compare the LPS/BMDMSC group to LPS/Saline and LPS/Fibroblast groups at each of given time points. Significance was set at a *P* value <.05.

## 3. Results

### 3.1. Characterization of Bone Marrow-Derived Mesenchymal Stem Cells

Enrichment of bone marrow-derived mesenchymal stem cells from crude bone marrow suspensions is achieved by selection for a plastic-adherent population that expresses neither hematopoietic nor endothelial cell surface markers but is positive for the expression of adhesion and stromal markers [3]. However, because a defined panel of unambiguous markers distinguishing MSC is lacking, a gold standard criterion for establishing MSC phenotype is a trilineage differentiation assay where the plasticity of MSC is confirmed by their ability to differentiate into adipocytes, osteocytes, and chondrocytes, on stimulation [16].

**Figure 1:**
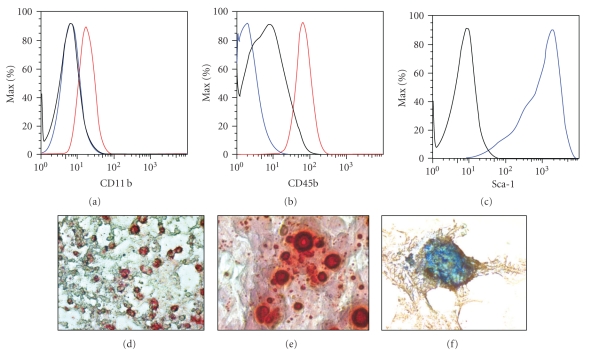
*Characterization of BMDMSC by flow cytometry and differentiation capacity. *Flow cytometry of BMDMSC demonstrated that cells did not express the hematopoietic markers, CD11b and CD45 ((a)-(b); blue; BMDMSC; grey; isotype control, red; alveolar macrophages). BMDMSC stained positive for Sca-1 ((c); blue; BMDMSC; grey; isotype control). In (d)–(f), cells were examined for differentiation capacity. Fat droplets in cell preparations incubated in adipogenic medium for 14 days were stained with Oil Red O (d). Cells incubated with osteogenic medium for 21 days were stained Alizarin red to detect Calcium (e). Cartilage pellets were stained with Alcian blue to detect mucopolysachharides, after 14 days in chondrogenic medium (f).

In the present study, we confirmed MSC phenotype by flow cytometry analysis and by differentiation assays. Analysis by flow cytometry demonstrated that the cells were uniformly negative for the hematopoietic markers, CD11b and CD45 (blue, BMDMSC). Cells stained positive for Stem cell antigen (Sca)-1 (Figures 1(a)–1(c)). Differentiation assays demonstrated that BMDMSCs retained their ability to differentiate into adipocytes, osteocytes, and chondrocytes for up to 10 passages (Figures 1(d)–1(e)). Thus, the cell population used in the present study represents a phenotype that is consistent with MSC.

### 3.2. Effect of BMDMSC Infusion on Endotoxin-Induced Inflammatory Response

We have previously reported that BMDMSC infusion attenuates the systemic inflammatory response to endotoxin [4]. In present study, we determined the effect of BMDMSC infusion on a select panel of pro-inflammatory cytokines to confirm that the anti-inflammatory effect of BMDMSC infusion was independent of LPS serotype administered (O55 : B5 in the present study versus O111 : B6 in [17]). Responses in LPS/BMDMSC group were compared to LPS/Saline group. 

**Figure 2:**
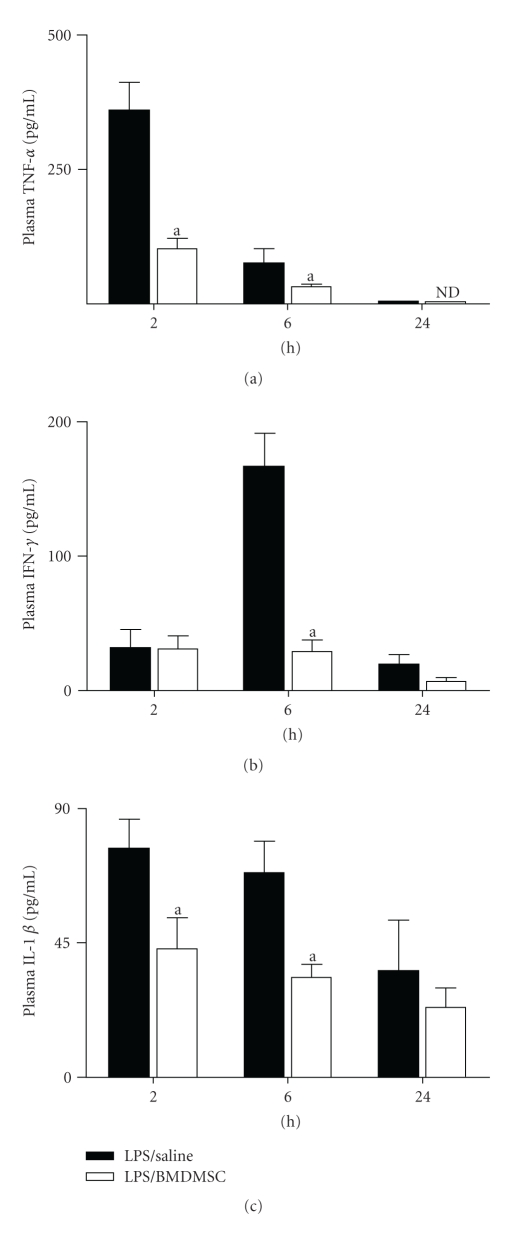
*Effect of BMDMSC infusion on endotoxin-induced inflammatory response. *C57BL/6J mice were treated with 1 mg/kg i.p LPS followed by i.v infusion of either 500,000 BMDMSC (LPS/BMDMSC), or 0.1 mL Saline (LPS/Saline) alone. At 2 hours, 6 hours, and 24 hours mice were sacrificed and plasma was collected for analysis of TNF-*α* (a), IFN-*γ* (b), and IL-1*β* (c). Data are expressed as means + SEM. ^a^Values in LPS/BMDMSC group significantly different from time-matched LPS/Saline group.

Plasma levels of TNF-*α* (Figure 2(a)), IFN-*γ* (Figure 2(b)), and IL-1*β* (Figure 2(c)) were determined at 2 hours, 6 hours, and 24 hours postendotoxin administration. Cytokine levels in Saline/BMDMSC group ranged from being non-detectable to <20 pg/mL at all time points and are not shown for clarity. 

Consistent with previous observations, results showed that BMDMSC infusion attenuated the acute inflammatory response to endotoxin. A significant decrease in plasma TNF-*α* levels was observed at 2 hours (Figure 2(a) (pg/mL); LPS/Saline, 360 ± 53; LPS/BMDMSC, 103.2 ± 19.2; *P* < .01) and 6 hours (LPS/Saline, 75 ± 28.2; LPS/BMDMSC, 32 ± 5; *P* < .05). Evaluation of plasma IFN-*γ* levels revealed a 5-fold decrease with BMDMSC infusion at 6 h (Figure 2(b) (pg/mL); LPS/Saline, 166.7 ± 24.1; LPS/BMDMSC, 29 ± 8.3; *P* < .001). 

Measurement of IL-1*β* levels demonstrated a 1.7-fold decrease in response to BMDMSC infusion at 2 hours (Figure 2(c) (pg/ml); LPS/Saline, 76.  4 ± 10; LPS/BMDMSC, 43 ± 10.2; *P* < .05) and a 2-fold decrease at 6 h (LPS/Saline, 68.6 ± 10.3; LPS/BMDMSC, 33.2 ± 4.6; *P* < .01). The decrease in TNF-*α*, IFN-*γ*, and IL-1*β* levels in LPS/BMDMSC group confirms the anti-inflammatory effects of BMDMSC in vivo.

### 3.3. Effect of BMDMSC Infusion on Endotoxin-Induced Depletion of Plasma GSH and Oxidation of GSH/GSSG Redox State

Having established that BMDMSC infusion induced anti-inflammatory effects in vivo, we next determined whether BMDMSC infusion was also associated with protection from perturbations in plasma GSH redox state. Because lung fibroblasts represent a primary, stromal-cell population originating from mesenchymal precursors, animals injected with LPS/Lung fibroblasts served as a second set of controls for these analyses.

**Figure 3:**
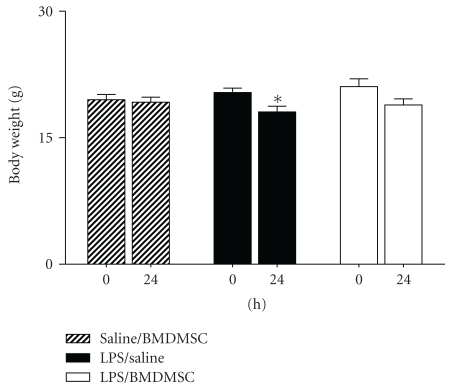
*Effect of BMDMSC infusion on endotoxin-induced weight loss.* C57BL/6J mice were treated with 1 mg/kg i.p LPS or with saline. LPS-treated animals were either given intravenous Saline (LPS/Saline) or BMDMSC (LPS/BMDMSC). Saline treated mice were given intravenous BMDMSC. Body weight was measured at 0 hours and 24 hours post treatment.

In previous studies we have found that endotoxin-induced weight loss occurs due to a decrease in food intake [7], and decreased food intake is an important determinant of plasma GSH and Cys levels [18]. In the present study, we determined loss in body weight 24 hours posttreatment in LPS/Saline group versus LPS/BMDMSC group. Mice receiving intraperitoneal saline followed by intravenous BMDMSC served as controls. As shown in Figure 3, mice treated with Saline/BMDMSC did not show a significant decline in body weight (body weight (g); 0 hours, 19.5 ± 0.6; 24 hours, 19.2 ± 0.6). As expected, LPS/Saline-treated animals lost a significant amount of body weight (0 hours, 20.3 ± 0.5; 24 hours, 18 ± 0.6; *P* < .05). However, body weight did not significantly decrease in mice treated with LPS/BMDMSC (0 hours, 21 ± 0.9; 24 hours, 19 ± 0.7; *P* = .05), suggesting protection due to BMDMSC infusion. It must be noted however that the effects of BMDMSCs on body weight are unlikely to be clinically significant. 

The dynamics of plasma GSH and Cys in response to endotoxin is previously described by our group [17]. In contrast to the sustained oxidation of GSH and Cys redox states until 24–48 hours that we previously reported; the present experiments show recovery of these thiol/disulfide couples by 24 hours. These differences can be ascribed to serotype differences in LPS. However, we cannot exclude the possibility that these differences may have also resulted, in part, from variations in the dose of endotoxic LPS delivered.

**Figure 4:**
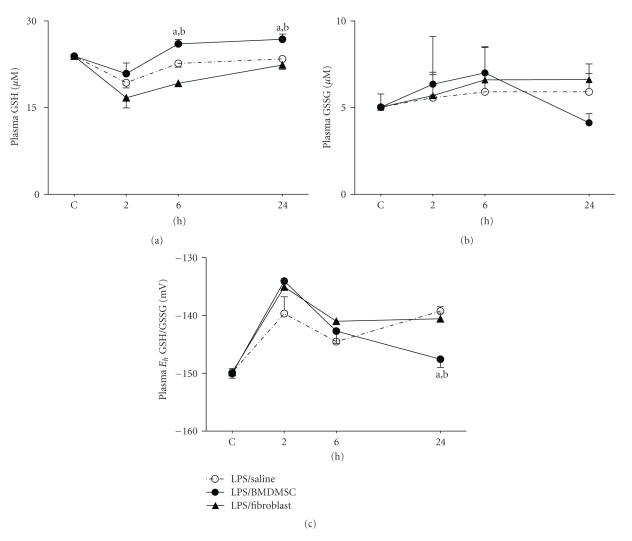
*Effect of BMDMSC infusion on endotoxin-induced depletion of plasma GSH and oxidation of GSH/GSSG redox state.* C57BL/6J mice were treated with 1mg/kg i.p LPS followed by i.v infusion of either 500,000 BMDMSC (LPS/BMDMSC), Lung fibroblasts (LPS/Lung fibroblast), or 0.1 mL Saline (LPS/Saline) alone. At 2 hours, 6 hours, and 24 hours mice were sacrificed and plasma was collected for HPLC analysis of GSH (a) and GSSG (b).   *E*_*h*_ GSH/GSSG (c) was calculated from the GSH and GSSG concentrations using the Nernst equation. Data are expressed as means + SEM. ^a^Values in LPS/BMDMSC group significantly different from time-matched LPS/Saline group. ^b^Values in LPS/BMDMSC group significantly different from time-matched LPS/Lung fibroblast group.

As seen in Figure 4(a), BMDMSC infusion had no effect on the acute decline in plasma GSH at 2 hours (plasma GSH (*μ*M), Control, 24 ± 0.2; LPS/Saline, 19.3 ± 0.9 (*P* < .001); LPS/BMDMSC, 20.8 ± 1.8), but protected against decline in GSH levels at 6 h (LPS/Saline, 22.7 ± 1.1; LPS/BMDMSC, 26 ± 0.7; *P* < .05), and 24 hours (LPS/Saline, 23.4 ± 0.4; LPS/BMDMSC, 26.8 ± 0.8; *P* < .05). Measurement of GSH levels in LPS/Lung fibroblast group revealed that GSH levels were significantly below LPS/BMDMSC group at 6 hours and 24 hours (LPS/Lung fibroblast; 6 hours, 19.2 ± 0.2; 24 hours, 22.3 ± 0.7; *P* < .01).

 Plasma GSSG did not show significant changes with treatment (Figure 4(b)). Calculation of GSH redox state using concentrations of GSH and GSSG revealed that *E*_*h*_ GSH/GSSG at 24 hours was, on average, 8 mV more reduced in LPS/BMDMSC group compared to LPS/Saline and LPS/Lung fibroblast group (Figure 4(c); E_h_ GSH/GSSG (mV), LPS/Saline, −139.2 ± 0.7; LPS/Lung fibroblast, −140.5 ± 1; LPS/BMDMSC, −147.6 ± 0.3; *P* < .01). The data show that BMDMSC protected against endotoxin-induced oxidation of GSH/GSSG redox state at 24 hours by improving GSH homeostasis.

### 3.4. Effect of BMDMSC Infusion on Endotoxin-Induced Depletion of Plasma Cys and Oxidation of Cys/CySS Redox State

**Figure 5:**
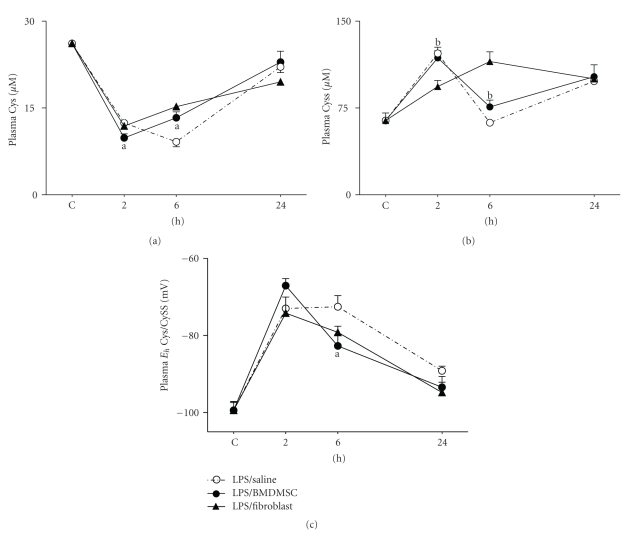
*Effect of BMDMSC infusion on endotoxin-induced depletion of plasma Cys and oxidation of Cys/CySS redox state.* C57BL/6J mice were treated with 1 mg/kg i.p LPS followed by i.v infusion of either 500,000 BMDMSC (LPS/BMDMSC), Lung fibroblasts (LPS/Lung fibroblast), or 0.1 mL Saline (LPS/Saline) alone. At 2 hours, 6 hours, and 24 hours mice were sacrificed and plasma was collected for HPLC analysis of Cys (a) and CySS (b). In (c), *E*_*h*_ Cys/CySS was calculated from the Cys and CySS concentrations using the Nernst equation. Data are expressed as means + SEM. ^a^Values in LPS/BMDMSC group significantly different from time-matched LPS/Saline group. ^b^Values in LPS/BMDMSC group significantly different from time-matched LPS/Lung fibroblast group.

Decrease in plasma Cys levels and oxidation of Cys/CySS redox state is an early event in endotoxin-induced ALI [7]. Results showed that BMDMSC infusion did not protect against the acute decline in plasma Cys levels at 2 hours (Figure 5(a); plasma Cys (*μ*M),Control, 26.1 ± 0.08; LPS/Saline, 12.4 ± 0.4; LPS/Lung fibroblast, 11.8 ± 1.7; LPS/BMDMSC, 9.8 ± 0.7; *P* < .01 compared to controls). Indeed, Cys levels in LPS/BMDMSC group were significantly lower than LPS/Saline group at 2 hours (*P* < .05). Despite this early decline, plasma Cys levels started to recover in LPS/BMDMSC treated group compared to LPS/Saline group at 6 hours (LPS/Saline, 9.1 ± 0.8; LPS/BMDMSC, 13.3 ± 1; *P* < .05). Cys levels in LPS/Lung fibroblast group also increased (15.2 ± 0.3; *P* < 0.05 compared to LPS/Saline group) and could not be distinguished from LPS/BMDMSC group at 6 hours. By 24 hours Cys levels normalized in all three treatment groups.

Plasma levels of the disulfide of Cys, CySS, increased significantly at 2 hours with endotoxin treatment and was not altered due to BMDMSC infusion (Figure 5(b); plasma CySS (*μ*M), Control, 64.2 ± 6.5; LPS/Saline, 122.2 ± 5.3; LPS/BMDMSC, 118.2 ± 6.1; *P* < .01 compared to controls). However, CySS levels in LPS/Lung fibroblast group were significantly decreased at 2 hours and increased at 6 hours prior to normalization at 24 hours (LPS/Lung fibroblast; 2 hours, 93.5 ± 5.3; 6 hours, 115.2 ± 8.4; *P* < .05 compared to LPS/Saline and LPS/BMDMSC group).

Despite no changes in CySS, preservation of plasma Cys by BMDMSC infusion resulted in *E*_*h*_ Cys/CySS being, on average, 8 mV more reduced, compared to LPS/Saline group at 6 hours (Figure 5(c); *E*_*h*_ Cys/CySS (mV), LPS/Saline, −72.5 ± 3; LPS/BMDMSC, −80.6 ± 2.4; *P* < .05). A similar magnitude of protection in *E*_*h*_ Cys/CySS was also observed in LPS/Lung fibroblast group (−79 ± 1.5; *P* < .05 compared to LPS/Saline). Thus, the data show that BMDMSC infusion resulted in a modest but significant preservation of plasma Cys and *E*_*h*_ Cys/CySS at 6 hours. However, this effect was not specific because similar responses were observed in the LPS/Lung fibroblast group. Together, the combined observations show that BMDMSC infusion resulted in sequential preservation of Cys and GSH redox states at 6 hours and 24 hours, respectively. The preservation of GSH was specific to BMDMSC infusion while Cys redox state was preserved with BMDMSC and Lung fibroblast infusion.

## 4. Discussion

The main finding of the present study is that infusion of BMDMSCs is associated with an increase in systemic Cys and GSH levels, which results in the preservation of Cys and GSH redox states during endotoxemia. These data are the first, to our knowledge, to demonstrate that BMDMSC infusion improves systemic thiol/disulfide redox status in vivo.

Several lines of evidence indicate that the biological thiol/disulfide redox environment dictates responses to inflammatory stimuli. Experimental manipulation of Cys and GSH levels alters cytokine production in vitro in mononuclear cells and [19] alters lymphocyte activation [20]. In vivo, oral pretreatment of mice with GSH and N-acetyl cysteine (NAC) attenuates LPS-induced TNF-*α* in the circulation and in the spleen [21]. Thus, Cys and GSH are critical determinants of cytokine production during activation of the immune system. However, in the present study we observe that the decrease in TNF-*α* and IL-1*β* at 2 hours precedes the increase in plasma Cys and GSH levels at 6 hours. This suggests that the improved homeostasis of these redox couples by BMDMSC infusion is not upstream to the decrease in proinflammatory cytokine levels. Nonetheless, because Cys and GSH redox systems are fundamental to basic cellular processes such as proliferation, differentiation, and apoptosis [22], the improvement in plasma Cys and GSH indices may have therapeutic effects in attenuating lung injury and/or facilitating repair. Thus, preservation of the thiol/disulfide redox environment may represent an additional therapeutic effect of BMDMSC infusion. 

The potential mechanisms by which BMDMSC infusion improves GSH homeostasis may involve increased efflux of Cys and GSH from tissues, increased recycling, and/or increased GSH synthesis. This can be mediated by the secretion of soluble growth factors by BMDMSCs, or by the interaction of BMDMSCs with host cells, or both. A candidate growth factor likely responsible for the redox modulatory effect of BMDMSCs is keratinocyte growth factor (KGF). BMDMSCs constitutively produce KGF [23], and studies in a murine model of allogenic bone marrow transplant show that subcutaneous infusion of KGF improves hepatic GSH levels [24]. Because 50%–80% of plasma GSH is dependent on efflux from the liver [25], increase in hepatic GSH could increase plasma GSH levels. Additionally, elevated levels of proinflammatory cytokines can adversely impact GSH homeostasis. In pulmonary endothelial cells, TNF-*α* decreases cellular GSH and increases GSSG levels [26]. Thus, BMDMSC-mediated decrease in plasma TNF-*α* and IL-1*β* levels at 2 hours could also have led to improved GSH homeostasis at subsequent time points. 

While improved homeostasis of plasma GSH is observed only in the BMDMSC group but not the lung fibroblast group, the dynamics of plasma Cys are similar in both groups. This would suggest that the improvement in plasma Cys is not a specific response BMDMSC infusion. Additional studies comparing stromal cells such as MSCs and lung fibroblasts with cell types from epithelial and endothelial lineages are needed to better delineate whether modulation of Cys homeostasis in vivo is a cell-type dependent or independent phenomenon. Nonetheless, because both the Cys and GSH redox systems are intimately linked to inflammation, repair, and regeneration, the preservation of both these redox systems by BMDMSCs is therapeutically significant. 

While the present observations are the first to demonstrate that BMDMSCs have redox modulatory effects in vivo, there is evidence supporting the antioxidant capacity of MSCs. For instance, Kim and colleagues have demonstrated that the antioxidant capacity of adipose tissue-derived MSC conditioned medium (ADCM) is comparable to 100 *μ*M ascorbic acid [27]. Furthermore, culturing tert-butyl hydroperoxide-treated dermal fibroblasts with ADCM improved cell viability, indicating that MSC-derived factors protect against oxidative injury. This study suggests that MSCs actively secrete antioxidant factors which may confer protection in the setting of inflammatory lung diseases such as ALI. However, studies in matrigel angiogenesis assay have demonstrated that direct contact of MSCs with endothelial cells (EC : MSC ratio; 1 : 1 to 1 : 3) led to increased ROS production resulting in endothelial cell apoptosis and ultimately to capillary degeneration [28]. A drop in cytotoxicity was observed when MSC numbers were decreased by an order of magnitude. These studies indicate that in vivo effects of MSCs may vary, depending on the MSC number and on the interacting cell population. Further studies investigating the effects of BMDMSC conditioned media on thiol/disulfide redox state will provide greater mechanistic insights into the action of BMDMSCs in vivo.

In conclusion, the present study demonstrates that in a murine model of endotoxin-induced inflammation, infusion of syngeneic BMDMSCs improved plasma Cys and GSH redox indices at 6 h and 24 hours, respectively. These studies are the first, to our knowledge, to demonstrate the antioxidant effects of BMDMSC in vivo. Further studies are needed to investigate the mechanistic basis for the improvement of thiol/disulfide redox status by BMDMSCs.
